# Continuous infusion of ceftazidime in critically ill patients undergoing continuous venovenous haemodiafiltration: pharmacokinetic evaluation and dose recommendation

**DOI:** 10.1186/cc3993

**Published:** 2006-02-13

**Authors:** Christophe Mariat, Christophe Venet, François Jehl, Sandrine Mwewa, Vesna Lazarevic, Eric Diconne, Nathalie Fonsale, Anne Carricajo, Stéphane Guyomarc'h, Régine Vermesch, Gérald Aubert, Roselyne Bidault, Jean-Claude Bertrand, Fabrice Zeni

**Affiliations:** 1 Service de Néphrologie, Hôpital Nord, CHU de Saint-Etienne, Saint-Etienne, France; 2 Service d'Urgences et de Réanimation, Hôpital Bellevue, CHU de Saint-Etienne, St Etienne, France; 3 Laboratoire de Bactériologie, Faculté de Médecine, Strasbourg, France; 4 Unité de Pharmacologie Clinique, Laboratoire Glaxo Wellcome, Marly Le Roi, France; 5 Service de Bactériologie, Hôpital Bellevue, CHU de Saint-Etienne, St Etienne France

## Abstract

**Introduction:**

In seriously infected patients with acute renal failure and who require continuous renal replacement therapy, data on continuous infusion of ceftazidime are lacking. Here we analyzed the pharmacokinetics of ceftazidime administered by continuous infusion in critically ill patients during continuous venovenous haemodiafiltration (CVVHDF) in order to identify the optimal dosage in this setting.

**Method:**

Seven critically ill patients were prospectively enrolled in the study. CVVHDF was performed using a 0.6 m^2 ^AN69 high-flux membrane and with blood, dialysate and ultrafiltration flow rates of 150 ml/min, 1 l/hour and 1.5 l/hour, respectively. Based on a predicted haemodiafiltration clearance of 32.5 ml/min, all patients received a 2 g loading dose of ceftazidime, followed by a 3 g/day continuous infusion for 72 hours. Serum samples were collected at 0, 3, 15 and 30 minutes and at 1, 2, 4, 6, 8, 12, 24, 36, 48 and 72 hours; dialysate/ultrafiltrate samples were taken at 2, 8, 12, 24, 36 and 48 hours. Ceftazidime concentrations in serum and dialysate/ultrafiltrate were measured using high-performance liquid chromatography.

**Results:**

The mean (± standard deviation) elimination half-life, volume of distribution, area under the concentration-time curve from time 0 to 72 hours, and total clearance of ceftazidime were 4 ± 1 hours, 19 ± 6 l, 2514 ± 212 mg/h per l, and 62 ± 5 ml/min, respectively. The mean serum ceftazidime steady-state concentration was 33.5 mg/l (range 28.8–36.3 mg/l). CVVHDF effectively removed continuously infused ceftazidime, with a sieving coefficient and haemodiafiltration clearance of 0.81 ± 0.11 and 33.6 ± 4 mg/l, respectively.

**Conclusion:**

We conclude that a dosing regimen of 3 g/day ceftazidime, by continuous infusion, following a 2 g loading dose, results in serum concentrations more than four times the minimum inhibitory concentration for all susceptible pathogens, and we recommend this regimen in critically ill patients undergoing CVVHDF.

## Introduction

The β-lactam antibiotics are known to exhibit little concentration-dependent activity. The maximal killing rate occurs when antibiotic concentrations have reached four to five times the minimum inhibitory concentration (MIC) for the causative pathogens [1], and higher concentrations do not significantly enhance the bactericidal activity. Conversely, an increase in the time for which free β-lactam serum levels are above the MIC is likely to improve antimicrobial activity. To optimize the use of these so-called time-dependent antibiotics, shortening the dose interval appears to be more critical than increasing the dose. These pharmacodynamic considerations have led to the proposition that continuous intravenous infusion may be the best way to administer β-lactams to maintain serum levels above the target concentration throughout the course of therapy [2-4].

Among β-lactams, ceftazidime is probably the most extensively studied during continuous intravenous infusion [5-12]. Although the clinical benefits of such a regimen are not yet definitely proven, ceftazidime tends to be widely administered by continuous infusion. Ceftazidime is a third-generation cephalosporin (molecular size 636.6 Da) with a broad spectrum of antibacterial activity (it is very active against *Pseudomonas aeruginosa*) and is commonly used in intensive care units. In patients with normal renal function, about 90% of ceftazidime is excreted unchanged by glomerular filtration [13]. In the setting of renal impairment, its pharmacokinetic profile is markedly altered and requires specific dosage adjustment [14-16]. It is well established [17-20] that a large proportion of administered ceftazidime is removed during continuous renal replacement therapies (CRRTs) as well as conventional haemodialysis, and the resulting pharmacokinetic modifications have been characterized in various patient groups, including critically ill patients undergoing continuous haemofiltration or haemodiafiltration [21,22]. Of note, these data were exclusively derived from studies in which ceftazidime was administered by intermittent infusions, and thus the findings cannot be extrapolated to continuous administration. The dosage for continuous infusion of ceftazidime required to achieve optimal therapeutic concentrations in the setting of CRRT is unknown. Our purpose was therefore to analyze the pharmacokinetics of ceftazidime administered by continuous infusion in critically ill patients during continuous venovenous haemodialfiltration (CVVHDF) and to identify the optimal dosage in this population.

## Materials and methods

### Study population

This prospective open-label study was conducted in a 15-bed intensive care unit of the teaching hospital of Saint-Etienne, France. Patients meeting the following criteria were eligible for inclusion in the study: age over 18 years; clinically suspected or proven bacterial infection; isolated or expected causative pathogen susceptible to ceftazidime; and acute renal failure requiring CVVHDF. Exclusion criteria were known allergy to ceftazidime or other β-lactams; use of ceftazidime within the 48 hours before enrollment; pregnancy, as determined by serum β-human chorionic gonadtotrophin testing; and residual glomerular filtration rate, measured by creatinine clearance, over 10 ml/min. The study protocol and the consent document were approved by the institutional review board, and written informed consent was obtained from each patient or a legally designated representative.

### Continuous venovenous haemodiafiltration

CVVHDF was performed using the PRISMA machine (Hospal, Meyzieu, France) equipped with a Multiflow 60 AN69HF 0.60 m^2 ^polyacrylonitrile hollow-fibre membrane. Vascular access was obtained by introduction of a 12 French, 16 or 20 cm double-lumen central venous catheter (Arrow, Reading, PA, USA) into a femoral vein. For all patients, operational characteristics of haemodialfiltration were set as follows: blood flow rate 150 ml/min; dialysate flow rate 1 l/hour; and ultrafiltration rate 1.5 l/hour. Substitution fluids were delivered according to a predilutional mode with a flow rate allowing 100–150 ml/min net ultrafiltration. Under this haemodiafiltration regimen, CVVHDF clearance of ceftazidime was predicted to be 32.5 ml/min [23].

Anticoagulation of the extracorporeal circuit was ordered at the discretion of the attending physician. If a patient was already undergoing CVVHDF, then the haemofilter was changed before the patient's inclusion in the study.

### Ceftazidime dosage and administration

After initiation of CVVHDF, all patients received a 2 g intravenous loading dose of cefatzidime (GlaxoSmithKline, Marly-le-Roi, France) infused over three minutes, via a central venous catheter different from that used for CVVHDF, and immediately followed by a 3 g continuous infusion over 24 hours. The ceftazidime dose was expected to provide serum antibiotic concentrations between 30 and 40 mg/l, and was selected according to the equation R_0 _= CL_tot _× Css, where R_0 _is the continuous administration rate, Css is the steady-state serum concentration of ceftazidime, and CL _tot _is the total clearance of ceftazidime (estimated using the equation CL_tot _= 0.693 × [volume of distribution/elimination half-life]). We anticipated a volume of distribution close to 0.30 l/kg based on data available in intensive care patients [24], and hypothesized a half-life of four hours, which is in keeping with the half-life found in a recent study [21] that examined similar patients and reported a haemofiltration clearance of ceftazidime in the same range as our predicted haemodiafiltration clearance.

Ceftazidime was continuously infused by the means of syringe infusion pump (Ivac Medical System, Basingstoke, UK) for at least 72 hours. Syringes with freshly dissolved antibiotic, were inserted every 16 hours. In case of CVVHDF clotting, the continuous infusion was stopped during change of haemofilter.

### Sample collection and assay

Arterial blood samples (7 ml) were collected from a radial catheter before the loading dose then at 3 (immediately after the 2 g bolus infusion), 15 and 30 minutes and at 1, 2, 4, 6, 8, 12, 24, 36, 48 and 72 hours. After collection, blood samples were immediately centrifuged (2000 *g *for 10 minutes) and stored at -80°C until analysis.

Seven samples (10 mL) were simultaneously drawn from the dialysate/ultrafiltrate outlet before ceftazidime infusion and at 2, 8, 12, 24, 36 and 48 hours. After collection, dialysate/ultrafiltrate samples were frozen and stored at -80°C until analysis. The concentrations of ceftazidime in the serum and dialysate/ultrafiltrate were assayed by high-performance liquid chromatography, employing the technique described by Jehl and coworkers [25].

Within-day and between-day coefficients of variability were both below 10% and the limit of quantification was 0.05 mg/l for serum and 1.0 mg/l for dialysate.

### Pharmacokinetic analysis

A noncompartmental model was applied. The following pharmacokinetic parameters were determined for each patient. The steady-state serum concentration of ceftazidime (mg/l) was calculated as the mean of the serum concentrations measured at 24, 36, 48 and 72 hours. The elimination half-life (hours) was calculated as ln2/k_e_, where k_e _is the apparent terminal elimination rate constant determined using least-squares regression analysis. The area under the concentration-time curve from time zero to 72 hours (mg/hour per l) was calculated using the linear trapezoidal summation method. The total clearance (ml/min) was calculated as R_0_/steady-state serum concentration of ceftazidime, where R_0 _is the continuous administration rate. Finally, the volume of distribution (l) was calculated as D/C_0_, where D is the loading dose and C_0 _the serum concentration at the end of the bolus infusion.

In addition, the following CVVHDF parameters were assessed. The sieving coefficient was calculated as C_D/UF_/C_S_, where C_D/UF _is the dialysate/ultrafiltrate concentration of ceftazidime and C_S _the time-corresponding serum concentration. The haemodiafiltration clearance (ml/min) was calculated as sieving coefficient × combined dialysate/ultrafiltrate flow rate [26]. The contribution (%) of haemodiafiltration clearance to total clearance of ceftazidime was calculated as follows: (haemodiafiltration clearance/total clearance) × 100.

### Statistical analysis

All calculations were made by programming pharmacokinetic and CRRT clearance equations into Microsoft Excel^® ^97 (Microsoft Corporation, Irvine, CA, USA). The same software program was used to compute mean and standard deviations for the various pharmacokinetic and haemodiafiltration parameters.

## Results

### Patient demographics

A total of seven patients were enrolled in the study from October 2000 to April 2002. All patients completed the scheduled period of pharmacokinetic sampling. None of the patients received ceftazidime before their enrollment in the study. The demographic characteristics of the patients are summarized in Table 1. The median time from admission to the intensive care unit to inclusion in the study was 15 days (range 2–30 days). All patients were male and were mechanically ventilated. They all had a suspected diagnosis of acute tubular necrosis as part of a multiple organ dysfunction syndrome and were already undergoing CVVHDF before enrollment, apart from patients 3 and 6. Residual diuresis during the study period was not significant in any patient except for patient 3, in whom urine output was above 1.5 l/day but with a measured creatinine clearance at inclusion of 5 ml/min. Infection was only documented in patients 1 and 3, in whom *Enterobacter aerogenes *(ceftazidime MIC = 1.5 mg/l; ETest^®^, AB BIODISK, Solna, Sweden) and *Escherichia coli *(ceftazidime MIC = 0.1 mg/l) were isolated, respectively. No adverse effects related to the drug or route of administration were reported during the study period.

### Pharmacokinetic parameters

**Figure 1:**
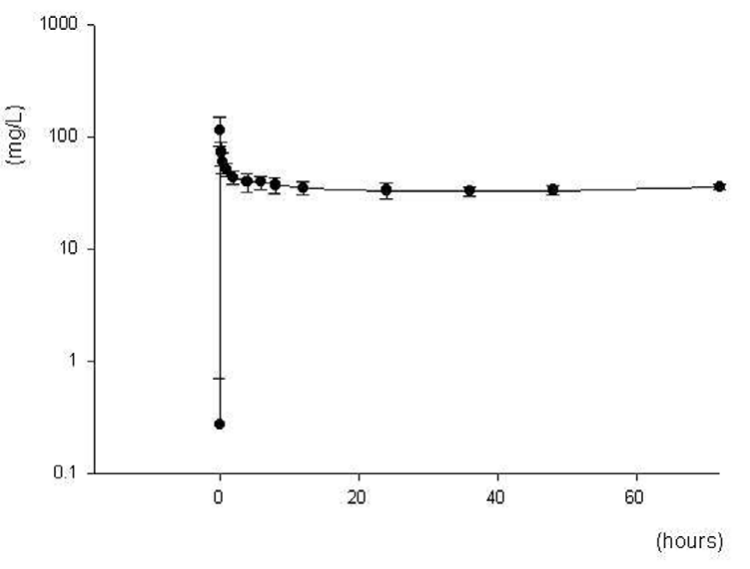
Ceftazidime concentration-time curve. Shown is a serum concentration-time curve for a 3 g/day continuous infusion of ceftazidime following a 2 g loading dose in seven critically ill patients undergoing continuous venovenous haemodiafiltration. Data points are the mean serum concentrations (± standard deviation) of ceftazidime measured at each time point during the 72-hour study period.

The evolution of mean serum concentrations over time is shown in Figure 1. In five of the seven patients ceftazidime concentrations were maintained above 30 mg/l for the entire study period. For patients 3 and 4, ceftazidime concentrations were below this threshold at 12, 24, 36 and 48 hours, and at 4 and 24 hours, respectively. However, for each patient the steady-state serum concentration of ceftazidime was within if not very close to the target concentration range of 30–40 mg/l, varying from 28.8 in patient 3 to 36.3 mg/l in patient 5. For the whole population the mean steady-state ceftazidime concentration was 33.5 ± 2 mg/l. Individual pharmacokinetic parameters are listed in Table 2. The mean elimination half-life was 4 ± 1 hours. The mean area under the concentration-time curve from time 0 to 72 hours, volume of distribution and total clearance were 2514 ± 212 mg/hour per l, 19 ± 6 l and 62 ± 5 ml/min, respectively.

### Continuous venovenous haemodiafiltration parameters

For all patients, blood flow rate, dialysate flow rate and ultrafiltrate rate were maintained as required by the study protocol throughout the pharmacokinetic sampling period. A mean of three haemofilter changes per patient was needed, ranging from no change (for patients 3 and 7) to six changes (for patient 4). The change procedure did not exceed 60 minutes, and neither did cessation of cefatazidime infusion in any patient.

**Figure 2:**
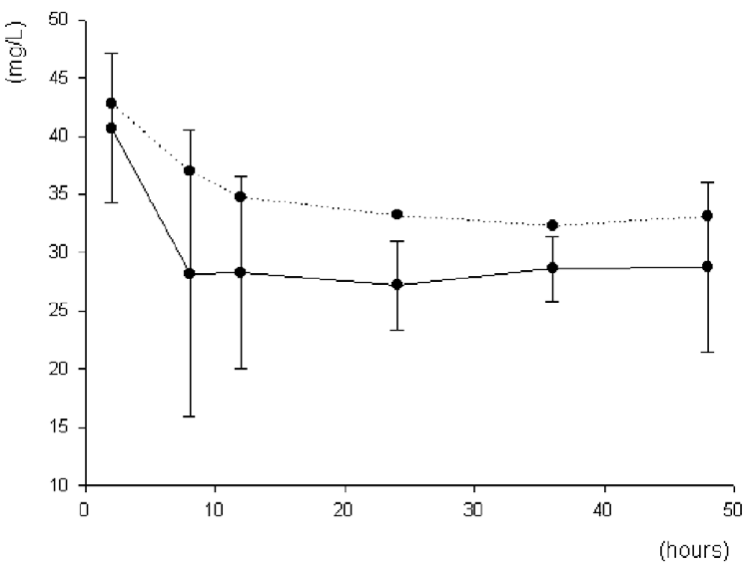
Ceftazidime removal by continuous venovenous haemodiafiltration. Shown is the removal of ceftazidime (administered as a 3 g/day continuous infusion) by continuous venovenous haemodiafiltration in seven critically ill patients. Data points connected by the continuous line are the mean ultrafiltrate/dialysate concentrations (± standard deviation) of ceftazidime; those connected by the dotted line are the corresponding mean serum concentrations of ceftazidime.

Individual ceftazidime haemodiafiltration clearances and sieving coefficients are given in Table 3. The mean haemodiafiltration clearance and sieving coefficient were 33.6 ± 4 ml/min and 0.81 ± 0.11, respectively, both indicating that ceftazidime was extensively cleared by CVVHDF. Of the ceftazidime total clearance, 55% was attributed to haemodiafiltration clearance. In addition, we observed constant removal of continuously infused ceftazidime by CVVHDF throughout the study (Figure 2).

## Discussion

In the management of critically ill patients with acute renal failure, it is important to characterize the pharmacokinetics and clearances of antibiotics during CRRT so that an appropriate dosing regimen can be selected that avoids both under-dosing and drug accumulation. To our knowledge, this study is the first one designed to investigate the pharmacokinetics of ceftazidime, administered by continuous infusion, in critically ill patients undergoing CVVHDF.

Like other studies conducted in intensive care patients [21,27], we found an increased volume of distribution for ceftazidime of 19 ± 6 l, as compared with values of less than 10 l in normal individuals [13]. In critically ill patients, this increased volume of distribution is thought to be due in large part to augmented total water (potentially accentuated by the kidney failure) combined with sepsis-related changes in fluid compartments, mainly increased capillary permeability [28]. The ceftazidime elimination half-life was 4 ± 1 hour, which is very close to the elimination half-life of 4.3 ± 0.6 hours previously reported in 12 patients treated with intermittent administration during CRRT [21]. This value is also similar to those in critically ill patients with normal renal function [7,27], emphasizing the effective removal of continuously infused ceftazidime by CVVHDF. In the present study the mean sieving coefficient was 0.81, varying from 0.66 to 0.94. These coefficient values are in the same range as those previously reported during intermittent administration [18,20,21]. As a result, the haemodiafiltration clearance was 33.6 ± 4 ml/min and contributed substantially (nearly 50% for each of the seven patients) to total clearance of ceftazidime. This result is consistent with the haemofiltration clearance of 32.1 ± 8 ml/min reported by Traunmüller and coworkers [21] in critically ill patients treated with 2 g ceftazidime every 8 hours, and undergoing partly comparable CRRT (haemofiltration alone, but with an enhanced ultrafiltration rate of approximately 3 l/hour and a mean blood flow rate of 143 ml/min).

Because extrarenal elimination pathways for ceftazidime are of minor importance, even in the case of renal impairment [14], non-CVVHDF clearance was expected to be negligible. Nevertheless, despite the presence of severe acute renal failure in our population, we noted that total clearance of ceftazidime was much higher than haemodiafiltration clearance. Although such a discrepancy between total and CRRT ceftazidime clearances was previously reported in critically ill patients [21], the explanation for this remains unclear. Degradation of ceftazidime has been described at room temperature in biological fluids [29,30], and this could therefore result in a falsely decreased haemodiafiltration clearance. However, in the present study *in vitro *degradation of ceftazidime is unlikely, given that all blood samples were carefully and promptly frozen after collection. Alternatively, one could argue that degradation of ceftazidime is also likely to occur *in vivo*, particularly in the case of prolonged elimination and increased temperature, and that this could enhance non-CVVHDF clearance. Interestingly, unexpected discrepancies between total and extracorporeal clearances in critically ill patients with no significant residual renal function have also been reported for cefepime [31,32]. Finally, significant adsorption of ceftazidime to synthetic membranes has not yet been investigated, and could account for increased total clearance as well.

However, despite this unexpected increased total clearance, the mean steady-state serum concentration of ceftazidime was within the targeted concentration range of 30–40 mg/l. Additionally, individual steady-state serum concentrations of ceftazidime, ranging from 28.8 to 36.3 mg/l, were rather homogenous bearing in mind the limited number of patients in our study and the variation in volume of distribution. Under CVVHDF, a 3 g/day continuous infusion of ceftazidime following a 2 g loading dose resulted in serum concentrations that were above four times the MIC of susceptible pathogens (MIC ≤4 mg/l) in all patients, and for the entire course of therapy. Moreover, the achieved serum concentrations were partially effective even against many intermediately susceptible strains (MIC ≤8 mg/l); ceftazidime could therefore be used as empirical therapy with this dosing regimen.

Only patient three regularly failed to achieve the expected steady-state serum concentration of ceftazidime. Although he experienced severe acute renal failure, this patient maintained a significant urine output throughout the study. Of note, in patients with severely impaired renal function (creatinine clearance between 2 and 10 ml/min) but still with diuresis, Leroy and coworkers [14] showed that about 25% of a single intravenous dose of ceftazidime can be recovered in urine after 24 hours. Therefore, significant urinary elimination of ceftazidime might have occurred in our patient. Whether this may explain the lower steady-state serum concentration of ceftazidime in this patient remains questionable, however, because ceftazidime concentrations in urine were not measured.

## Conclusion

Our findings confirm that, in critically ill patients undergoing CRRT, the pharmacokinetics of ceftazidime remain unchanged when it is infused continuously. Using similar operational characteristics for CVVHDF, we recommend administration of a continuous infusion of 3 g ceftazidime over 24 hours, following an initial 2 g loading dose, in critically ill patients with severe acute renal failure.

## Key messages

• 	CVVHDF effectively removes continuously infused ceftazidime, with a mean sieving coefficient of 0.81 and a mean CVVHDF clearance of 33.6 ml/min.

• 	In critically ill patients with severe acute renal failure treated with CVVHDF and receiving a continuous infusion of cefatzidime, extrarenal clearance of ceftazidime is substantial and accounts for about 50% of total clearance of the drug.

• 	As compared with intermittent administration, continuous infusion of ceftazidime does not significantly affect the pharmacokinetic profile of the drug in critically ill patients receiving CVVHDF.

• 	We recommend a 2 g loading dose of ceftazidime followed by 3 g/day continuous infusion in critically ill patients undergoing CVVHDF.

## Abbreviations

CRRT = continuous renal replacement therapy; CVVHDF = continuous venovenous haemodiafiltration; MIC = minimum inhibitory concentration.

## Competing interests

The authors declare that they have no competing interests.

## Authors' contributions

CM, CV, ED, NF, AC, SG, RV, GA, J-CB and FZ participated in designing the study, collecting the samples and writing the manuscript. FJ, SM, VL and RB designed and conoducted the pharmacokinetic analysis, and helped to draft the manuscript.

## Figures and Tables

**Table 1:** Demographic characteristics of the seven study patients

Patient	Age (years)	Weight (kg)	Height (cm)	SAPS II score	Urine output (ml/24 hours)^a^	Residual glomerular filtration rate (ml/min)^b^
1	65	76	170	42	300	<1
2	43	70	195	46	50	<1
3	51	76	165	28	1,650	5
4	71	89	178	64	0	0
5	46	61	183	65	150	<1
6	68	83	168	63	300	<1
7	68	88	163	64	100	<1

^a^Mean daily urine output during time of pharmacokinetic sampling. ^b^Creatinine clearance measured at the inclusion; if necessary, continuous venovenous haemodiafiltration was stopped before enrollment to assess creatinine clearance. SAPS, Simplified Acute Physiologic Score.

**Table 2:** Individual and mean pharmacokinetic parameters of ceftazidime

Pharmacokinetic parameters	Css	AUC_0–72_	CLtot	Vd	T_1/2_
Patient 1	35.2	2625.0	59.2	19.0	3.7
Patient 2	34.3	2655.0	60.8	12.4	2.8
Patient 3	28.8	2146.0	72.3	17.7	2.8
Patient 4	32.5	2372.0	64.1	29.4	5.3
Patient 5	36.3	2795.0	57.5	24.1	5.0
Patient 6	33.6	2471.0	62.0	13.7	2.6
Patient 7	34.2	2536.0	61.0	15.8	3.0
Mean	33.5	2514.3	62.4	18.9	3.6
Standard deviation	2.4	211.8	4.8	6.0	1.1

AUC_0–72 h_, area under the concentration time-curve from time zero to 72 hours (mg/hour per l); CL_tot_, total clearance of ceftazidime (ml/min); Css, steady-state serum ceftazidime concentration (mg/l); T_1/2_, elimination half-life (hours); Vd, volume of distribution (l).

**Table 3:** Individual and mean continuous venovenous haemodiafiltration parameters

Parameter	Patient							Mean	Median	SD	Min	Max
						
	1	2	3	4	5	6	7					
Sc	0.94	0.91	0.87	0.93	0.66	0.69	0.7	0.81	0.84	0.11	0.66	0.94
CLhdf	38.5	37.2	35.8	37.8	27.7	28.6	29.2	33.6	34.7	4	27.7	38.5

CLhdf, haemodiafiltration clearance of ceftazidime (ml/min); Sc, sieving coefficient.
